# The Impact of Participation in the Olympics on Post-olympic Performance in Professional Ice Hockey Players

**DOI:** 10.3389/fspor.2020.00076

**Published:** 2020-07-03

**Authors:** Emily Bremer, John Cairney

**Affiliations:** ^1^Faculty of Kinesiology and Physical Education, University of Toronto, Toronto, ON, Canada; ^2^School of Human Movement and Nutrition Sciences, University of Queensland, St Lucia, QLD, Australia

**Keywords:** National Hockey League, fatigue, ice hockey, Winter Olympics, elite sport

## Abstract

The 2014 Sochi Winter Games were the last Winter Olympics where NHL players were allowed to compete. One explanation for prohibiting NHL players from participating in the Winter Olympics is a perceived negative impact on their performance post-Olympics, owing to the additional fatigue of participating. The purpose of this study was to explore whether participation in the 2014 Sochi Winter Games negatively impacted individual NHL player performance post-Olympics. A database was constructed to examine NHL player points per game played as the performance outcome pre- and post- the 2014 Winter Olympics during the 2013–2014 NHL season. Three multilevel models were fitted with post-Olympics points per game as the outcome. Model 1 examined the effect of Olympic minutes played, controlling for pre-Olympics points per game. Model 2 introduced player position (forward vs. defense) and model 3 included an interaction between player position and Olympic minutes played to determine if there were differential effects of Olympic participation on post-Olympic performance by position. The results show that Olympic minutes played did not have a significant main effect on post-Olympics performance (*p* > 0.10). There was a significant interaction between Olympic minutes played and playing position whereby forwards who played a higher number of minutes in the Olympics scored fewer points per game post-Olympics than forwards playing fewer Olympic minutes. The magnitude of this effect, however, was quite small [*b* (SE) = −0.003 (0.001), *p* = 0.03]. These findings suggest that the effect of Olympic playing time on individual player performance post-Olympics is minimal.

## Introduction

Although Pierre De Coubertin, founder of the modern Olympic movement, would most certainly disagree with the practice, allowing so-called professional athletes to compete in the Olympics has become standard practice over the past few decades. For example, professional basketball and tennis players routinely compete in the summer games. Professional athletes involved in winter sports are also allowed to compete in the modern games. In 1995, an agreement between the governing professional body [National Hockey League (NHL)], the amateur associations, and the International Olympic Committee (IOC), was reached and professional ice hockey players were allowed to compete for the first time at the 1998 winter games, more than 71 years following the introduction of the winter Olympics to the program (Lapointe, 1997). Very early on however, controversy surrounded the introduction of professionals, with the NHL in particular complaining about the impact the winter games had on the professional schedule and the threat of injury to players. While it is not uncommon for professional sport organizations to send their players to international competitions, the case of the NHL and the Winter Olympics is unique in that the games occur in the middle of the regular season. This interruption in regular season play raises concern about the potentially negative impact to team performance that arises from sending the leagues' top players to an elite competition (Longley, 2012).

One of the more prominent concerns over allowing professional ice hockey players to compete in the Winter Games is related to the impact it may have on post-Olympic performance. Longley (2012) coined the term “fatigue theory” to describe the effect: he hypothesized that players sent to the Olympics would experience post-Olympic fatigue, a result of both the mental and physical demands at playing at a level of international competition, which in turn would negatively impact team performance. At the team level, the effect is exacerbated by the fact that professional teams often send not only their best players, but multiple players to the Games. In fact, the top teams, not surprisingly, often have multiple players who would be eligible to compete for their countries. A team whose best players return fatigued from the competition may suffer in terms of performance, at least in the short run following resumption of the professional season. In the worst-case scenario, a player may receive a season-ending injury as a result of play, as in the case of John Tavares, whose knee injury sustained during the 2014 Olympics ended his professional playing season with the New York Islanders. Of course, the fatigue may itself be further influenced by factors such as the location of the Games: sending players to a foreign country in a different time zone may further fatigue, not to mention change in diet, sleep and training regimes. Also, the position a player plays on the Olympic team (e.g., back-up Goalie vs. first line defensemen or forward) and how deep the team goes into the competition would all potentially contribute to greater fatigue levels.

To our knowledge, only two studies have tested the fatigue theory in this context. Longley (2012) examined team performance, specifically goal differential (goals for minus goals against), using archival data for the Winter Olympics from 1998 to 2010. He found that the more players sent from a single team, the worse the team performed post-Olympics. Because the top performing teams send more players on average than the bottom performing teams, a competitive imbalance in the League post-Olympics resulted. Cairney et al. (2015) also examined goal differential using archival data from 1998 to 2014. Unlike Longley however, Cairney et al. (2015) used growth curve modeling to examine changes over time in goal differential, pre- and post-Olympics. Moreover, they examined the fatigue effect for each Games separately, to assess whether the location of the Games also influenced the post-Games team performance. Their results showed that the number of players sent had a negative impact on goal differential, but only during the 1997–98 NHL season (Cairney et al., 2015).

Fatigue theory postulates a mechanism at the individual player-level, which in turn affects team performance, especially under conditions where multiple players experience the fatigue of competition and the combined influence of their fatigue produces overall decrements in team performance. While both Longley (2012) and Cairney et al. (2015) provide some evidence in support of this effect, it remains to be seen whether participation in the Olympics actually impacts individual-level player performance. In both studies, goal differential, the main outcome, is an indicator of team performance rather than individual performance *per se*; therefore, a direct test of fatigue theory on individual performance has yet to be tested. Moreover, neither of these studies adequately examined the question of exposure related to the fatigue hypothesis. In other words, fatigue is a function of exposure to play: the more an athlete plays, the greater the fatigue and chance of increased injury and/or diminished performance (assuming there is inadequate time for recovery). Just as teams do not send the same number of players to the Games, not all players who attend the Olympics have equal exposures. Teams that go deeper into competition will have more opportunity for playing time; different positions and where the player is in the line-up (e.g., first vs. third line player) will also impact time played as well coaching decisions (e.g., matching lines to opponents to create offensive and defensive advantages). While Longley (2012) statistically adjusted for whether the team medaled in the Games, this is at best a rough proxy for estimating total exposure. A more direct test of the fatigue theory must involve an examination of the impact of minutes played on post-Olympic performance. Moreover, it is likely that player position (e.g., defense vs. offense) will further impact the effect of minutes played, given variability in play factors such as distance and speed covered while on the ice, puck control, and body contact (Green et al., 1976).

In this paper, we examined whether participation in the 2014 Sochi Winter Games, the last Winter Olympics where NHL players were allowed to compete, negatively impacted individual player performance post-Olympic Games. To do this, we examine whether the number of minutes played in the Games negatively impacts player performance post-Olympics. Given that the main exposure (minutes played) may not be the same across different positions, and that the impact of time played on post-Olympic performance may vary by position, we tested for an interaction of player position by minutes played in predicting post-Olympic performance.

## Method

### Data Extraction

In order to answer our research questions, we used a within subject, longitudinal design, constructing a database from official records. As the primary focus concerned the impact of participation in the Olympics on post-competition, regular season play, a within subject design without a control group was deemed to be the most appropriate for initial exploration of the research questions. This allowed us to ascertain if the duration of participation impacted post-Olympic play among a cohort of NHL players selected. A database was constructed to examine NHL player points per game played as the performance outcome pre- and post- the 2014 Winter Olympics during the 2013–2014 NHL season. First, Olympic team rosters were extracted from the official website of the International Ice Hockey Federation (IIHF; http://sochi2014.iihf.com/men/statistics/), identifying all NHL players who participated in the 2014 Olympics, excluding goalies. Second, we extracted player level statistics from the official website of the NHL (http://www.nhl.com/stats/player) for all regular season games for two periods: pre-Olympics (October 1, 2013–February 9, 2014) and post-Olympics (February 25, 2014 to April 13, 2014). The database included: NHL team, position, number of games played, goals, assists, and total points. Finally, we extracted the total number of playing minutes in the Olympics for each player from the official website of the IIHF. The data were publicly available and not restricted for use in this context.

### Dependent Measure

The average points per game played post-Olympics was used as the dependent variable. This was calculated by dividing the total points post-Olympics by the number of games played post-Olympics.

### Independent Variables

Our primary independent variable was minutes played at the Olympics. The NHL team a player played on was treated as a categorical variable, position was treated as a binary variable (0 = defense, 1 = forward). Finally, we also included the average points per game played during the pre-Olympics period to account for prior performance.

### Statistical Analysis

Three multilevel models were fitted with post-Olympics points per game as the outcome. Model 1 examined the effect of Olympic minutes played, controlling for pre-Olympics points per game. Model 2 introduced player position (forward vs. defense) and model 3 added an interaction between player position (forward vs. defense) and Olympic minutes played to determine if there were differential effects of Olympic participation on post-Olympic performance by position. A random intercept for the player's NHL team was included in each of the three models. All other variables were included as fixed effects and no random slopes were included in the models. Analyses were conducted in SPSS 25 (IBM Corporation, 2017) and R (R Core Team, 2019) using the *lme4* package (Bates et al., 2015).

## Results

The database included 128 players, representing all 30 NHL teams. Four players were, however, excluded from the analysis as they were injured during the Olympics and did not play in any NHL games post-Olympics. Two of these injured players were the only players at the Olympics from the Florida Panthers. Thus, the final analysis included 124 players (62.1% forwards) from 29 NHL teams.

Table 1 shows the average points per game by position, pre- and post-Olympics, as well as the number of minutes played during the Olympics. As we would expect, forwards scored more points per game on average than defensive players, and this was true before and after the Olympics. As well, defensive players played more minutes on average than offensive players during the Games. There was also a slight reduction in points per game for forwards after the Olympics, and a very small increase for defensive players.

**Table 1:** Difference in key variables by player position.

**Variable**	**Forward (*n* = 77) Mean (SD)**	**Defense (*n* = 47)Mean (SD)**	***p*-value**	**Effect sizeCohen's *d* (95% CI)**
Pre-olympics PPG	0.69 (0.25)	0.40 (0.20)	<0.001	1.24 (0.82–1.65)
Post-olympics PPG	0.67 (0.29)	0.41 (0.23)	<0.001	0.98 (0.59–1.37)
Olympic minutes played	75.89 (23.79)	84.27 (31.61)	0.096	−0.31 (−0.68–0.06)

*PPG, Points per Game Played*.

**Figure 1:**
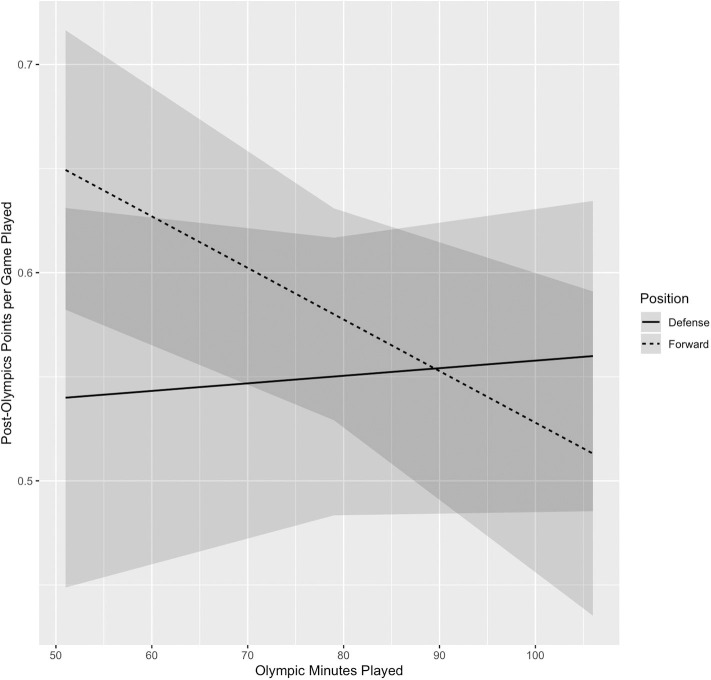
Interaction between Olympic minutes played and playing position on post-Olympics points per game.

Results from the three multilevel models (Table 2) indicate that there was not a significant main effect of Olympic minutes played on post-Olympics performance. Significant main effects were present for pre-Olympics points and position, whereby more points pre-Olympics and playing forward had a positive effect on post-Olympic performance. There was also a significant interaction between Olympic minutes played and playing position (Model 3). Forwards who played a higher number of minutes in the Olympics scored fewer points per game post-Olympics than forwards playing fewer Olympic minutes. In contrast, there was virtually no effect of minutes played on post-Olympic performance for defensemen. Figure 1 depicts the interaction between Olympic minutes played and player position.

**Table 2:** Multilevel models of the effect of Olympics minutes played and playing position on post-Olympics points per game.

	**Model 1**		**Model 2**		**Model 3**	
	**Estimate (SE)**	***p*-value**	**Estimate (SE)**	***p*-value**	**Estimate (SE)**	***p*-value**
**FIXED PARTS**						
Intercept	0.19 (0.07)	**<0.01**	0.18 (0.07)	**<0.01**	0.06 (0.09)	0.47
Pre-olympics points per game played	0.81 (0.07)	**<0.001**	0.78 (0.08)	**<0.001**	0.79 (0.08)	**<0.001**
Olympic minutes played	−0.001 (0.001)	0.10	−0.001 (0.001)	0.15	0.0004 (0.001)	0.69
Forward position			0.03 (0.04)	0.54	0.25 (0.11)	**0.03**
Olympic minutes played * forward position					−0.003 (0.001)	**0.03**
**RANDOM PARTS**						
Team (intercept) variance (SD)	0.004 (0.06)		0.004 (0.06)		0.003 (0.05)
Residual variance (SD)	0.04 (0.19)		0.04 (0.19)		0.04 (0.19)
N_teams_	29		29		29
ICC_teams_	0.10		0.09		0.08
**MODEL STATISTICS**						
AIC	−36.8		−35.2		−37.7
BIC	−22.7		−18.3		−17.9
Observations (*N*)	124		124		124
Marginal R^2^/conditional R^2^	0.53/0.57		0.53/0.57		0.55/0.58

*Bold values indicate statistical significance*.

## Discussion

According to our analysis of the 2014 Winter Games, we found no significant effect of Olympic time played on post-Olympic points per game, but there was a significant interaction between minutes played and position. This suggests that there was a differential effect of the Olympics by position, with forwards who played more at the Olympics producing fewer points post-Olympics. This effect, however, is almost negligible with less than a 0.2-point difference between those forwards who played above-average minutes vs. below-average minutes. This in fact is similar to the team-level analyses conducted by Longley (2012) and Cairney et al. (2015) in that they also found significant, but small effects of player participation on post-Olympic performance. Regardless of the outcome or level of analysis (individual player vs. team performance), the emergent evidence suggests any impact is likely small, thereby refuting the contention that there is a significant negative cost in relation to sending NHL players to compete in the middle of the season. At the same time, our data revealed a season-ending injury prevalence of 3.1% (4 out of 128 players). These four players included John Tavares (New York Islanders; knee injury), Henrik Zetterberg (Detroit; back injury), Aleksander Barkov (Florida; lower body injury), and Tomas Kopecky (Florida; concussion). None of these players were able to return to regular season play following their injury, although Zetterberg did return for two games in the playoffs (Rosen, 2017). Risk of injury is always a concern and although the prevalence of season-ending injuries according to our data was small, the increased playing time accompanies an increased risk of injury (Smith et al., 1997).

Although professional ice hockey players have participated in the Winter Olympics since 1998, in 2017, the NHL announced that it would not allow players to participate in the 2018 Winter Olympics citing a number of concerns, most notably, a failure to reach a monetary agreement with the IOC (Rosen, 2017). Therefore, one may question the value of such a study, beyond historical interest, given that at least at present, professional ice hockey players are not allowed to compete in the Winter Olympics. Debate at the time the NHL withdrew from the Games centered in part on the ethics of denying athletes the chance to compete for their countries. The NHL indicated it would reconsider instating players in the future if a suitable agreement with the IOC could be reached. Therefore, even though at present professional ice hockey players are not allowed to compete in the Winter Games, it is important to continue to examine stated reasons of concern over participation as it is unlikely the debate about letting these players compete is over.

As noted in our methods, we chose a within subject cohort design to examine the impact of participation before and after participation in the Olympics. As such, there is no comparison or control group. While we argue this is an appropriate design for the research questions explored, follow-up analysis could be done comparing pre- and post-Olympic play among players who participated in the games, vs. those who did not. One of the arguments for a competitive disadvantage created by sending players to the Olympics is that for players who do not attend the Games, they effectively receive a 2-week holiday that allows for rest and recuperation from injuries sustained prior to commencement of the Games (Longley, 2012; Cairney et al., 2015). Comparing players who did, and did not, compete would allow for a test of this relative competitive (dis)advantage at the team level. Establishing a relevant comparison group however will be challenging. Player selection to play in the Olympics is based on multiple factors such as skill level, position, and Nationality. Finding a matched control based on all of these factors could prove extremely difficult. Nevertheless, given that we found a small effect among forwards, it would be interesting to examine whether similar position players who did not attend the Games experience a late season boost in performance, owing to the recovery they received while the Olympics were being played. This will of course depend on exactly what players are doing during this break to balance the stress/fatigue state (Bird, 2011), and whether or not the time off is adequate to promote recovery from fatigue and minor injury that will positively impact performance on return to play. Whether this varies by position would also be an interesting question to further explore.

In conclusion, our results provide initial evidence that player-level performance is not significantly affected by participation in the Olympics—while we saw a differential effect by player position, the impact on performance was minimal. This finding supports the team-level analyses conducted by Longley (2012) and Cairney et al. (2015), demonstrating minimal effect on post-Olympic performance. While the NHL's decision to stop sending its players to the Olympics was primarily financial; the additional playing time and subsequent fatigue was also cited as a deterrent to participation. The results from this study, however, suggest that the effect on player performance is minimal.

## Data Availability Statement

The data underlying this study are third-party and therefore cannot be published alongside the article. These data are publicly available through the official website of the International Ice Hockey Federation (http://sochi2014.iihf.com/men/statistics/) and the official website of the NHL (http://www.nhl.com/stats/player).

## Ethics Statement

Ethical review and approval was not required for the study on human participants in accordance with the local legislation and institutional requirements. Written informed consent for participation was not required for this study in accordance with the national legislation and the institutional requirements. Written informed consent was not obtained from the individual(s) for the publication of any potentially identifiable images or data included in this article.

## Author Contributions

EB generated the database, conducted the data analyses, and drafted the initial manuscript. JC conceptualized the study, supervised the design, and execution of all phases of the study. All authors reviewed and approved the final manuscript.

## Conflict of Interest

The authors declare that the research was conducted in the absence of any commercial or financial relationships that could be construed as a potential conflict of interest.
